# Optimization of HS-SPME/GC-MS Method for Determining Volatile Organic Compounds and Sensory Profile in Cocoa Honey from Different Cocoa Varieties (*Theobroma cacao* L.)

**DOI:** 10.3390/molecules29133194

**Published:** 2024-07-05

**Authors:** Manuela B. Nascimento, Lívia R. Amorim, Marcos A. S. Nonato, Mariana N. Roselino, Ligia R. R. Santana, Adriana C. R. Ferreira, Frederico M. Rodrigues, Paulo R. R. Mesquita, Sergio E. Soares

**Affiliations:** 1 Faculty of Pharmacy, Federal University of Bahia, Campus Ondina, Salvador 40170-115, BA, Brazil; manuelabarreto_n@hotmail.com (M.B.N.); livia.amorim07@gmail.com (L.R.A.); marcos.nonato@ufba.br (M.A.S.N.); mariana.roselino@ufba.br (M.N.R.); 2 Agricultural Technological Center of the State of Bahia, Ondina, Salvador 40170-110, BA, Brazil; fredericomr@hotmail.com (F.M.R.); prrmesquita@gmail.com (P.R.R.M.); 3 Department of Life Sciences, State University of Bahia, Cabula, Salvador 41195-001, BA, Brazil; lrsantana@uneb.br; 4 Cocoa Innovation Center, Santa Cruz State University, Salobrinho, Ilhéus 45662-200, BA, Brazil; adriana_reis@pctsb.org

**Keywords:** cocoa honey, multivariate optimization, volatile organic compounds, sensory profile, descriptive analysis, consumer acceptance

## Abstract

This study aimed to develop an analytical method using HS-SPME/GC-MS to determine the volatile organic compound (VOC) profiles and evaluate the sensory attributes of cocoa honey from four cocoa varieties (CCN51, PS1319, SJ02, and Parazinho). Using a multivariate factorial experimental design, the HS-SPME/GC-MS method was optimized to determine the VOC profiles. Twenty previously trained tasters participated in the ranking descriptive analysis, while 108 consumers participated in the acceptance and purchase intention tests. A total of 84 volatile organic compounds were identified from various chemical classes, including acids, alcohols, aldehydes, esters, ketones, monoterpenes, oxygenated monoterpenoids, sesquiterpenes, and oxygenated sesquiterpenoids. Palmitic acid was the compound found in the highest concentration in all varieties (5.13–13.10%). Multivariate analysis tools identified key compounds for differentiation and grouping of the samples. The results revealed that the variety significantly influenced both the VOCs’ concentrations and sensory profiles. The CCN51, PS1319, and SJ02 varieties exhibited the highest diversity of VOCs and sensory attributes. Notably, the SJ02 and CCN51 varieties demonstrated superior acceptability and purchase intention, with means ranging from 7.21 and 7.08 to 3.71 and 3.56, respectively. These results indicate their potential as promising sources of cocoa honey for the food industry.

## 1. Introduction

Cocoa (*Theobroma cacao* L.) is a fruit native to Central America that is cultivated in several countries. World production is led by the Ivory Coast, with approximately 61% of production, placing Brazil in sixth position with 4.4% [1]. In Brazil, although Bahia was once the pillar in cocoa production, today the state of Pará is the leader, with production of 150,000 tons in 2023 [2]. Originally, there were three varieties of cocoa cultivated, the Criollo, Forastero, and Trinitario, the latter being a hybrid of the first two varieties. Currently, advances in genetic engineering and subsequent processing of beans have led to the use of disease-resistant clones derived from these original varieties. CCN51, PS1319, and SJ02 are hybrids resulting from the genetic improvement of Trinitario cacao, while Parazinho comes from the Forastero variety, which has not undergone genetic improvement [3]. Overall, more than 1000 different cocoa varieties have been described [4].

However, the extraction of this fruit generates residues that have recently been the subject of studies aimed at new industrial applications. These residues include cocoa shell, pulp, and cocoa honey. In this context, cocoa honey is an opaque yellow, mucilaginous liquid with a bittersweet flavor, separated from the pulp surrounding the cocoa beans by simple cold extraction before fermentation starts [5]. Cocoa honey gets its name from its rheological characteristics, such as its viscosity and sweet flavor, which resemble bee honey, although it does not originate from beekeeping. The literature shows that cocoa honey is a by-product with physicochemical characteristics similar to the original pulp, such as its reducing sugar content (8.63 g 100 g^−1^), low ash content (approximately 0.2%), low lipid content (0.25 g 100 g^−1^), and acid pH ranging from 2.76 to 3.78 [6,7].

The main uses of cocoa honey include the production of alcoholic beverages, syrups, jellies, and liqueurs, often made by cooperatives and artisanal food producers [5]. Some of these include kefir-based drinks [8], mixed açaí jelly and cocoa honey [9], and functional drink cocoa honey kombucha [10]. Additionally, some patents employed cocoa honey as a sugar substitute in edible ice cream and chocolate [11], and in the production of craft beer [12]. In addition, volatile organic compounds (VOCs) are crucial constituents for developing the aroma of cocoa honey. Among the methods used to analyze these VOCs, headspace solid-phase microextraction coupled with gas chromatography–mass spectrometry (HS-SPME/GC-MS) stands out as an efficient and versatile technique. This method involves the adsorption of volatile and semi-volatile organic compounds from a matrix by a fused silica fiber or a fiber coated with a non-volatile polymer, without direct contact with the sample. These compounds are then injected into a gas chromatography system for analysis (GC) [13].

Since VOCs can affect the sensory quality of cocoa honey, it is important to analyze not only the compounds themselves but also their correlation with consumer perception and acceptance. The sensory quality of a food product promotes consumer loyalty in an increasingly demanding and diversified market. Therefore, it is necessary to evaluate its color, flavor, aroma, and consistency properties. Sensory methods are used to evaluate these properties, which can be of the discriminative, descriptive, subjective, or affective types [14]. In this context, ranking descriptive analysis (RDA) was applied by Richter et al. [15] for chocolate pudding and by Silva et al. [16] for chocolate formulations to rank the intensity of each sensory attribute in increasing order. RDA offers advantages such as reduced time and costs, as it requires fewer sessions and smaller quantities of the product compared to other techniques.

Although cocoa is an ancient fruit and the extraction of cocoa honey has existed for decades, there are, to our knowledge, no available data on the evaluation of volatile organic compounds or the sensory profile of the different varieties of cocoa honey explored in this work. Thus, this study aimed to develop an analytical method using HS-SPME/GC-MS to identify the volatile organic compounds present in different varieties of cocoa honey, in addition to evaluating their sensory profile through descriptive and affective analysis.

## 2. Results and Discussion

### 2.1. HS-SPME Optimization

The first parameter evaluated in this study was the fiber coating used to extract volatile organic compounds from cocoa honey samples through univariate analysis. According to Brokl et al. [17], the polarity of the fibers depends on the coating material, which determines the interaction and extraction capacity of the compounds present in the sample. This, in turn, may be reflected in different chromatographic peak areas.

**Figure 1:**
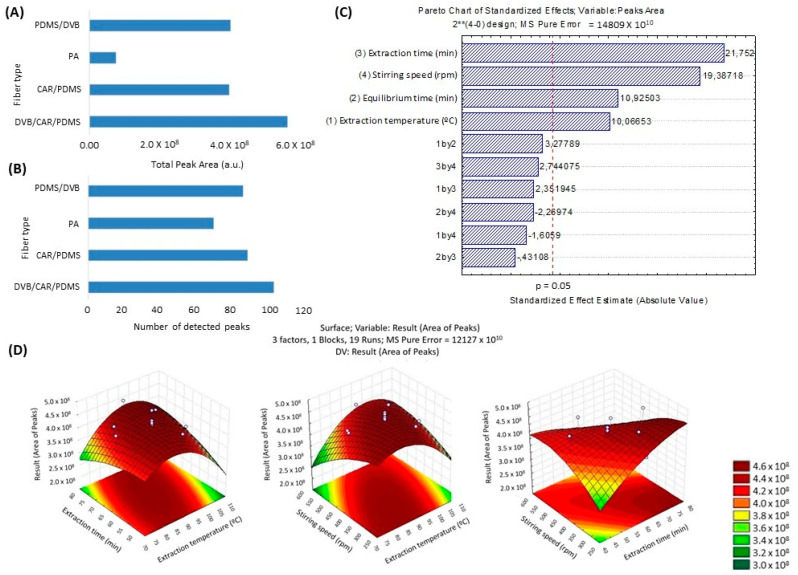
Influence of the type of HS-SPME fiber coating on the extraction efficiency of VOCs in cocoa honey samples considering total peak area (**A**) and number of detected peaks (**B**). Pareto chart of standardized effects of the 2^4^ factorial design for total chromatographic peak area (**C**). Response surface obtained by central composite using the quadratic model in the optimization of the conditions (temperature T, °C, time t, min, and stirring speed, rpm) for extraction of the volatiles in cocoa honey by HS-SPME (**D**).

Four types of fibers were used under the same conditions: sample amount (10 mL), extraction temperature (60 °C), equilibration time (15 min), extraction time (30 min), and stirring speed (100 rpm). The total peak area and the number of peaks detected in the extractions performed were considered for comparing the four types of fibers. The extractions were performed in triplicate, and the reproducibility (% RSD) showed an error of less than 20%. According to the results, the DVB/CAR/PDMS fiber extracted the largest peak area (Figure 1A) and obtained the highest number of peaks (Figure 1B).

The four fibers tested had the following order of extraction efficiency: DVB/CAR/PDMS > CAR/PDMS > PDMS/DVB > PA. It is evident that the mixed-phase fibers achieved higher extraction efficiency. A total of 103 chromatographic peaks were detected with the DVB/CAR/PDMS fiber, 89 with the CAR/PDMS fiber, 86 with the PDMS/DVB fiber, and 70 with the PA fiber. Therefore, the DVB/CAR/PDMS fiber was chosen for the optimization tests of the other parameters of the HS-SPME technique for extracting VOCs from cocoa honey. DVB/CAR/PDMS is considered one of the most efficient SPME coating types, as it combines three different materials: DVB for strong retention of aromatic compounds and polar analytes, CAR for small volatile molecules, and PDMS for non-polar compounds. This type of fiber has been previously used to evaluate volatile organic compounds in cocoa and its by-products [18,19].

A full 2^4^ factorial design was performed to evaluate the influence of temperature, extraction time, equilibration time, and stirring speed, as detailed in Table S1. The Pareto chart shows that all four parameters were significant and influenced the efficiency of the HS-SPME method (Figure 1C). However, to reduce the number of experiments and not excessively increase the total extraction time, the equilibration time was fixed at 20 min.

Thus, a central composite multivariate design was used to determine the optimal values of temperature, extraction time, and stirring speed for achieving greater extraction efficiency by HS-SPME. A central composite design was constructed using the experimental levels that provided the best response as the center point, involving 19 experiments and 5 replicates at the central point (Table S2).

Figure 1D shows the response surfaces obtained through the central composite design, indicating that the optimal conditions were within the experimental domain. The empirical regression equation for the response (total area) of HS-SPME extraction, dependent on extraction temperature (A), extraction time (B), and stirring speed (C), is shown in Equation (1):

Response = 4.30 × 108 − 5.64 × 106 A + 2.43 × 106 B − 1.07 × 107 C − 2.52 × 107 A^2^ + 1.31 × 107 AB + 2.09 × 107 AC − 5.65 × 106 B^2^ − 1.52 × 107 BC − 8.12 × 106 C^2^ (1)

The determination coefficient (R^2^) of the quadratic regression model was 0.4056, and the lack of fit was 0.0014 (*p* < 0.05), indicating that the sum of the areas can be explained but the model has low predictive accuracy. Therefore, it was necessary to experimentally validate the optimal points predicted by the model. Table S3 shows that the optimal values for temperature (85 °C), extraction time (78 min), and stirring speed (171 rpm) obtained through the central composite design for the HS-SPME method, after optimization, provided a better response compared to similar conditions for these parameters.

In the study by Rojas et al. [20], the extraction conditions for HS-SPME of cocoa liquor of the CCN51 variety were optimized using a 33 experimental design, considering the factors of fiber type, temperature, and extraction time. According to the design, the best conditions for extracting VOCs were an extraction time of 50 min, a temperature of 60 °C, and a DVB/CAR/PDMS fiber. The identified compounds included aldehydes, ketones, alcohols, pyrazines, esters, terpenes, acids, and lactones, with acetic acid, 2-propanol, and 3-ethyl-2,5-dimethyl-pyrazine being the compounds with the highest concentrations.

### 2.2. Characterization of Volatile Organic Compounds

The volatile profile of cocoa honey samples was evaluated to identify VOCs associated with different cocoa varieties. Table 1 shows all VOCs identified among the different varieties, their linear retention index (LRI) on the HP-5 MS capillary column, and the average relative composition (n = 9) of each compound in the sample from each variety. A total of 84 volatile organic compounds were identified, and 16 were confirmed using the standard. So far, this is the first study evaluating the VOC profile of cocoa honey obtained from different cocoa varieties (*Theobroma cacao* L.). A similar study identified 56 volatile organic compounds in the cocoa pulp of different varieties (CATIE-R1, CATIE-R4, CATIE-R6, ICS-95 (T1), and PMCT-58) harvested at different maturation stages in Costa Rica [4].

In this study, the samples differed in the number of volatile organic compounds detected in each variety (Table 1). In general, the SJ02 and PS1319 varieties had the highest number of volatiles (72 and 70 compounds, respectively), followed by Parazinho and CCN51 (67 and 63 compounds, respectively). Among the detected compounds, the chemical classes included volatile acids (6), alcohols (8), aldehydes (13), esters (21), ketones (5), monoterpenes (10), oxygenated monoterpenes (7), sesquiterpenes (5), oxygenated sesquiterpenes (4), and others (4) across the four varieties.

Six compounds were found in the four varieties within the group of volatile acids. Palmitic acid was present in the highest concentration across all varieties; other acids, such as dodecanoic, tetradecanoic, and pentadecanoic, were also detected. Palmitic acid is a fatty acid found in cocoa butter, cocoa, and chocolate products. These results align with findings from other studies comparing unroasted cocoa beans from different geographic origins, where palmitic acid was a major component, and dodecanoic acid, tetradecanoic acid, and pentadecanoic acid were present in lower concentrations [21]. Although cocoa pulp contains low amounts of fat, unsaturated fatty acids are likely present in cocoa pulp [22]. These results may explain the presence of these fatty acids in the cocoa honey samples, as cocoa honey is a by-product of cocoa, obtained by pressing its pulp.

The variety PS1319 was characterized by the highest amounts of alcohols (28.72%), with higher contents of 2-pentanol and 2-heptanol. In contrast, the samples of the varieties Parazinho (25.74%), CCN51 (24.79%), and SJ02 (20.75%) contained lower levels of these compounds. According to other studies, 2-pentanol is widely identified in cocoa pulp [23]. The 2-heptanol is an aromatic compound that imparts a citrus flavor (similar to lemongrass), a fresh aroma, and sweet and floral notes when found in foods [24]. Also noteworthy in this work is the presence of 2-nonanol in the varieties CCN51, PS1319, and SJ02. In the work of Valdeci et al. [18], 2-nonanol was produced during the fermentation of cocoa beans. According to Chetschik et al. [25], it is a desirable compound commonly found in high-quality cocoa. A high alcohol content is desirable in cocoa products because it imparts sweet and floral notes.

**Table 1:** Mean relative composition (%) of VOCs identified in cocoa honey from different cocoa varieties (*Theobroma cacao* L.).

Group	Compound	Odor Description	tR (min)	LRI_EXP_ ^a^	LRI_LIT_ ^b^	CCN51	PS1319	SJ02	Parazinho
Acids									
1	octanoic acid	Sweet, cheese, oily, fatty [26,27]	38.971	1183	1185	0.20	0.01	0.02	0.09
2	nonanoic acid *	Green, fatty [26]	42.820	1271	1272	0.42	0.03	0.09	0.22
3	dodecanoic acid *	Rancid, fatty, metal [26]	52.850	1562	1562	0.56	0.83	0.62	0.70
4	tetradecanoic acid *		58.444	1758	1759	0.42	0.27	0.38	0.64
5	pentadecanoic acid *		61.002	1855	1857	2.73	0.14	0.20	0.27
6	palmitic acid *		63.814	1961	1960	13.10	5.13	8.27	7.72
Alcohols									
7	2-pentanol	Fruity [28]	5.050	687	685	2.23	2.43	3.52	4.12
8	2-heptanol	Citrus, fresh, lemon-grasslike [24], sweet, citrusy [26,27,29]	20.987	901	901	0.93	2.06	0.37	0.05
9	1-heptanol *		27.384	980	975	-	-	0.03	-
10	2-ethyl-1-hexanol		30.912	1031	1030	-	-	0.01	-
11	2-nonanol	Fruity [4,30]	34.677	1099	1098	0.50	2.26	0.11	0.00
12	1-dodecanol *		50.001	1470	1469	0.13	0.09	0.12	0.33
13	1-tetradecanol		56.075	1672	1672	0.12	0.08	0.07	0.18
14	1-hexadecanol *		61.485	1874	1879	0.29	0.15	0.14	0.41
15	(*Z*)-9-octadecen-1-ol		67.671	2054	2060	2.54	0.74	1.27	1.91
Aldehydes									
16	furfural	Bread, almond, sweet [28]	12.882	832	835	0.22	0.16	0.30	0.15
17	benzaldehyde *	Bitter [24,27], candy, almond, burnt sugar [27]	25.925	964	961	0.70	0.27	0.38	0.42
18	octanal *	Citrus-like [25]	29.290	1000	1000	0.25	0.20	0.26	0.04
19	benzeneacetaldehyde		31.370	1040	1049	0.43	-	-	0.17
20	(*E*)-2-octenal *		32.322	1057	1062	0.62	0.31	0.33	0.27
21	decanal		39.977	1202	1202	0.39	0.24	0.43	0.23
22	2,5-dimethylbenzaldehyde		40.300	1210	1208	0.85	-	0.63	0.27
23	(*E*)-2-decenal		42.353	1260	1263	1.71	0.86	1.45	0.97
24	(*E*)-cinnamaldehyde		42.723	1269	1266	0.14	0.08	0.14	0.16
25	(*E*,*E*)-2,4-decadienal	Tallow, fatty [25]	44.505	1313	1314	1.28	1.01	0.49	0.55
26	2-undecenal	Tallowy, sweet [25]	46.256	1361	1363	1.24	0.65	1.00	0.31
27	(*E*)-2-dodecenal		49.756	1463	1462	0.14	0.13	0.13	0.03
28	tetradecanal		54.256	1608	1608	0.07	0.05	0.06	0.05
Esters									
29	1-methylbutyl acetate	Fruity [4]	14.810	852	843	11.92	15.66	23.87	14.18
30	pentyl propanate		24.939	952	952	0.11	0.08	0.08	0.06
31	(*Z*)-3-hexen-1-ol acetate		28.241	989	988	0.03	-	0.05	0.01
32	hexyl ethanoate		30.097	1016	1016	0.02	0.01	0.01	0.04
33	2-heptyl acetate	Fruity [30]	31.688	1046	1047	5.95	10.89	3.78	1.24
34	2,3-butanediyl diacetate	Floral [4]	33.437	1077	1065	0.07	0.11	0.07	0.16
35	1,3-butylene diacetate		35.729	1120	1124	2.32	3.29	-	6.26
36	benzyl ethanoate	Floral, jasmine [27,31] fresh [27]	38.044	1166	1170	0.27	0.14	0.09	0.19
37	gardenol	Sweet, fruity [29]	39.581	1194	1186	1.19	2.16	2.72	6.87
38	neryl acetate		40.979	1227	1221	0.18	0.34	0.32	0.19
39	3-phenyl-1-propyl acetate		46.512	1368	1373	-	0.14	0.17	0.43
40	decyl acetate		48.626	1429	1418	-	0.10	0.02	-
41	(*E*)-cinnamyl acetate		49.067	1442	1452	-	0.10	-	0.10
42	hexyl benzoate		53.573	1585	1580	0.95	1.63	0.35	0.16
43	methyl jasmonate		55.351	1647	1644	0.66	0.30	0.30	0.52
44	methyl (3-oxo-2-[(2*Z*)-2-pentenyl]cyclopentyl)acetate		56.221	1677	1684	0.44	0.18	0.21	0.43
45	benzyl benzoate		58.590	1763	1763	0.31	0.08	0.05	-
46	2-ethylhexyl salicylate *		59.673	1803	1807	0.40	0.59	0.55	0.70
47	isopropyl myristate		60.116	1820	1823	-	0.04	-	0.04
48	methyl palmitate		62.652	1918	1921	0.02	0.03	0.04	0.04
49	propyl palmitate		68.400	2068	2065	0.07	0.07	0.08	-
Ketones									
50	2-heptanone *	Fruity [31], cheese-like [24], flowery [26], pear, grape, brandy [27]	19.334	889	889	0.69	0.97	0.37	0.48
51	acetophenone	Sweet, almond, flowery [31], must-like [24,26], almond	32.560	1062	1062	0.15	0.38	0.64	2.00
52	2-nonanone	Flowery, fruit, musty [27]	34.142	1089	1090	0.96	1.75	0.49	0.30
53	geranyl acetone *		49.333	1450	1452	0.21	0.30	0.24	0.26
54	nerylacetone		51.706	1524	1535	-	0.10	-	-
Monoterpene									
55	7-endo-ethenyl-bicyclo[4,2,0]-oct-1-ene		30.206	1018	1029	-	-	0.04	-
56	*β*-cymene		30.291	1019	1025	-	0.01	-	-
57	D-limonene *	Citrus, orange, sweet [31]	30.517	1024	1027	0.04	0.12	0.12	0.05
58	(*Z*)-ocimene	Floral [30]	31.350	1039	1039	-	-	0.34	-
59	3-carene		31.823	1048	1040	-	0.21	0.18	-
60	linalol *	Flowery, lavender, rose [28]	34.557	1096	1099	1.21	2.45	2.76	0.78
61	allo-ocimene		36.158	1129	1131	-	0.07	-	-
62	(*E*,*E*)-cosmene		36.170	1129	1130	0.05	-	0.15	0.07
63	(*Z*)-geraniol	Floral, rose, fruity [31]	42.100	1254	1254	0.78	0.70	0.74	0.49
64	piperitone		43.796	1294	1285	0.04	0.05	-	0.11
Oxygenated monoterpene									
65	(*Z*)-linalool oxide	Sweet, nutty [24,31]	32.992	1069	1068	0.48	0.22	1.04	0.94
66	(*E*)-linalool oxide	Floral [24]	33.831	1084	1081	0.83	0.45	1.64	0.84
67	epoxylinalol	Floral [4]	38.528	1175	1163	-	0.03	0.02	0.16
68	*α*-terpineol		39.240	1188	1188	0.52	0.77	0.69	0.31
69	*β*-phellandrene-8-ol		40.446	1214	1215	-	-	0.03	0.02
70	(*E*)-*β*-damascenone		47.040	1382	1385	0.20	0.13	0.58	0.34
71	*α*-ferulene		50.106	1474	1484	-	0.02	0.02	-
Sesquiterpene									
72	elixene		48.885	1437	1445	0.03	-	0.03	0.12
73	*δ*-guaiene		50.469	1484	1493	-	0.03	0.03	-
74	(*Z*,*E*)-farnesene		50.579	1488	1486	-	0.15	0.05	0.13
75	germacrene D		51.666	1523	1529	0.13	-	-	0.13
76	phytane		59.423	1793	1795	0.11	0.07	0.05	0.15
Oxygenated sesquiterpene									
77	7,8-dihydro-β-ionone		48.852	1436	1433	-	0.04	0.01	-
78	*γ*-eudesmol	Woody [28]	54.947	1632	1633	0.70	0.67	0.59	0.95
79	bulnesol	Pepper-like [32]	55.569	1654	1659	0.33	0.38	0.42	0.54
80	thunbergol		66.710	2036	2047	-	0.80	-	-
Others									
81	2,2,6-trimethyl-6-vinyltetrahydropyran		26.926	975	971	-	0.03	0.01	-
82	2-pyridinemethanamine		31.839	1049	1054	0.12	-	-	0.03
83	benzothiazole		40.643	1219	1224	0.03	-	0.03	0.01
84	*γ*-decalactone	Fruity, peach [31]	49.822	1465	1463	-	-	-	0.08

* Identification confirmed by comparison with mass spectra and retention times of analytical standards. ^a^ LRI_EXP_ = linear retention index to the standard of n-alkanes (C_7_ to C_30_) obtained in an HP-5 MS capillary column. ^b^ LRI_LIT_ = linear retention index published in the literature. Odor description references are listed in the references section [4,24,25,26,27,28,29,30,31,32].

Thirteen aldehydes were detected in cocoa honey, with notable compounds including (*E*)-2-decenal, (*E*,*E*)-2,4-decadienal, and 2-undecenal. Depending on their concentrations in the final product, the presence of these compounds is often associated with lipid degradation and can impart either unpleasant or pleasant notes. The pleasant notes conferred by aldehydes include sweet and bitter flavors, such as almond and cherry aromas [33].

Among the 22 esters identified in the cocoa honey varieties, 1-methylbutyl acetate, 2-heptyl acetate, and gardenol stand out. Also known as 2-pentanol acetate, 1-methylbutyl acetate has a fruity odor and was found in the study by Hegmann et al. [4] in cocoa fruit pulp. The compounds 2-heptyl acetate and gardenol were identified in cocoa pulp from Colombia [23].

Five ketones were found in the four cocoa honey varieties, with the major compounds being 2-heptanone, acetophenone, and 2-nonanone. The 2-heptanone has a penetrating fruity odor and has been identified during the spontaneous fermentation of fine-flavor Trinitario cocoa beans, although is more closely associated with the cocoa bean, despite being observed in the first few hours of fermentation [30]. Furthermore, 2-heptanone and acetophenone were identified in the study by Hegmann et al. [4] when evaluating volatile organic compounds in the cocoa pulp of different varieties. Hegmann et al. [4] and Rottiers et al. [30] suggested that 2-nonanone contributed to the fruity aroma, as found in their studies of VOCs in pulp and cocoa beans, respectively.

Among the ten identified monoterpenes, linalool was the most predominant compound in the four varieties. A similar result was observed in the study by Valdeci et al. [18], where linalool had the highest compound concentration in cocoa beans during fermentation. According to Owusu et al. [34], linalool can be found in both the cocoa pulp and in the cotyledons of the bean in its glycosidic form. It has a floral aroma that is considered the main aroma component in high concentrations of “noble flavor” cocoas, such as Criollo and Arriba (a Forastero cocoa subtype). Furthermore, D-limonene was found in all four varieties in lower concentrations. Valdeci et al. [18] also identified D-limonene in cocoa beans and pulp during fermentation.

The (*Z*)-linalool and (*E*)-linalool oxides are among the seven oxygenated monoterpenes found in the four varieties of cocoa honey. The compound (*E*)-linalool oxide has a floral aroma, and (*Z*)-linalool oxide has a sweet and flowery aroma. Both were identified in the study by Hegmann et al. [4] when evaluating the cocoa pulp of different varieties, seasons of the year, and maturation stages. The result is similar to that found in the research conducted by Valdeci et al. [18] when evaluating the VOCs of cocoa beans and pulp that underwent fermentation.

Thus, the VOCs in cocoa honey predominantly showed higher levels of esters, acids, and alcohols, respectively. This result aligns with several studies related to cocoa and its derivatives [18,19,23,29]. According to the findings of this study, several authors agreed that the volatile organic compounds of cocoa and chocolate are formed during fermentation, where the cocoa cotyledon absorbs the aromatic compounds of the pulp during the fermentation process by mass transfer and in the almond roasting process by the Maillard reaction [35]. The presence of several VOCs was observed in this research, which are also present in the almond, cocoa pulp, chocolate, and cocoa honey. However, considering that the cocoa honey did not undergo the fermentation process, it is evident that a significant portion of the volatile organic compounds were present in all cocoa components, regardless of whether they undergo fermentation.

### 2.3. Multivariate Analysis

To evaluate the diverse profiles of volatile organic compounds in cocoa honey varieties, different multivariate analysis tools were applied, such as principal component analysis (PCA) and hierarchical cluster analysis (HCA) coupled with the heat map. PCA is a multivariate modeling technique that can reduce the dimensionality of data and determine the principal components. Additionally, it represents the data based on their similarities and differences [36].

**Figure 2:**
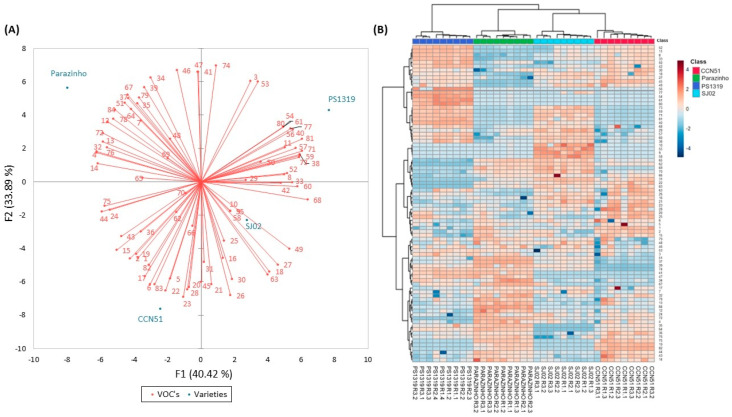
Principal component analysis (PCA) of the volatile components found in different varieties of cocoa honey (**A**). Average of 9 replicates per treatment. Numbers in red correspond to the VOC’s listed in Table 1. Hierarchical cluster analysis dendrogram associated with the heat map of the VOC profiles of the four cocoa honey varieties (**B**). The color scale represents the variation in the relative concentration of VOCs in the samples, from low (blue) to high (red).

Figure 2A shows the biplot graph of PCA scores and loadings, containing samples and volatile organic compounds from cocoa honey of different cocoa varieties. The PCA visualization indicated that the PS1319 and Parazinho varieties exhibited distinct compound profiles, while the CCN51 and SJ02 varieties were more closely related. Compounds such as 1-dodecanol, elixene, and 2,3-butanediyl diacetate significantly contributed to the discrimination of the VOC profile in the Parazinho variety, whereas the variety PS1319 was discriminated by compounds 2-heptanone, *β*-cymene, and decyl acetate. For the CCN51 variety, notable compounds included octanoic acid, (*Z*)-9-octadecen-1-ol, and hexadecanoic acid, while for the SJ02 variety, 2-ethyl-1-hexanol, 7-endo-ethenyl-bicyclo [4,2,0]-oct-1-ene, and decanal stood out. In the PCA conducted by Calva-Estrada et al. [37], differences in the volatile profile were also observed in samples of North American chocolates of the Trinitario and Criollo genotypes.

Figure 2B shows the dendrogram obtained through hierarchical cluster analysis (HCA) associated with the heat map. In this dendrogram, the samples were grouped based on the similarity of their VOC profiles, clearly showing the formation of four distinct clusters. The cluster comprising samples of the PS1319 variety (dark blue) exhibited a profile more distinct from the other three varieties, whereas the CCN51 and SJ02 varieties displayed greater similarities in their volatile profiles. The dark and light blue clusters (PS1319 and SJ02) consist of samples with higher concentrations of monoterpenes and esters compared to the green and red groups (Parazinho and CCN51). This correlation can also be evident in Table 1, which presents the average relative composition of the identified VOCs.

Thus, it was observed that it is possible to discriminate among the four varieties of cocoa honey based on their VOC profiles, which exhibit different compositions in each variety. Although no other works in the literature have employed a heat map to evaluate the VOC profiles in cocoa honey, this multivariate analysis technique has been applied to other matrices. Calva-Estrada et al. [37] demonstrated that the heat map enabled discrimination of chocolates based on the origin and variety of cocoa when studying the volatile compound profiles of commercial dark chocolates from different cocoa bean varieties (CA100, CV74, CM70, TM66, and NE60) from Latin America.

### 2.4. Sensory Evaluation of Cocoa Honey

To conduct the ranking descriptive analysis (RDA), 20 pre-trained tasters participated. These tasters generated a list of 15 descriptive attributes: yellowish color, greenish color, acid aroma, sweet aroma, cocoa aroma/cocoa pulp, fruity aroma, mint/refreshing aroma, floral aroma, acidic flavor, sweet flavor, cocoa flavor/cocoa pulp, fruity taste, astringent taste, viscosity, and smoothness. Table 2 presents the sum of the rankings based on the intensity of each attribute obtained across the cocoa honey varieties.

The results of the RDA revealed a significant difference (*p* ≤ 0.05) between the sensory attributes of the samples from the four varieties of cocoa honey under the experimental conditions, indicating that the tasters’ training was adequate. Varieties showed significant differences (*p* ≤ 0.05) among themselves in the yellow color attribute, except for varieties PS1319 and Parazinho. For the green color attribute, the CCN51 and Parazinho varieties significantly differed from the other varieties, exhibiting a more intense green color, as determined by the Friedman test at a 5% probability level. A similar result was found by Nascimento et al. [38] when analyzing the color of cocoa honey from the varieties CCN51, PS1319, SJ02, and Parazinho. In terms of aroma, the CCN51 and PS1319 varieties were considered the most acidic, differing significantly from the other two varieties. The sweet aroma was similar between the CCN51 and SJ02 varieties but significantly different between the PS1319 and Parazinho varieties. The cocoa aroma differed between the CCN51/PS1319 and SJ02/Parazinho varieties. The fruity and minty/refreshing aromas were more intense in the CCN51 and PS1319 varieties. The floral aroma presented similar results in the SJ02 and Parazinho varieties.

In terms of the acid taste attribute, the CCN51 and Parazinho varieties differed significantly from the other varieties, exhibiting a higher acidity content. This could be attributed to the acid aroma, with the CCN51 variety showing one of the highest intensities. The PS1319 and SJ02 varieties were perceived as the sweetest by the tasters. The low intensity of the sweet flavor in the CCN51 and Parazinho varieties may be overshadowed by the high intensity of the acid flavor perceived by the tasters. A similar pattern was observed by Valdeci et al. [18] in chocolates produced after spontaneous fermentation of the TSH565 cocoa clone.

The cocoa and fruity tastes differed only in the Parazinho variety, with the lowest intensity, while the astringent taste was more pronounced in the Parazinho and CCN51 varieties. This is attributed to the inherent astringency of fresh cocoa beans [39], and since cocoa honey undergoes cold pressing before fermentation, there is no reduction in astringency. Eskes et al. [40], in their evaluation of the sensory characteristics of cocoa pulp based on genetic variation, noted that the CCN51 variety was characterized as astringent and acidic. According to Boza et al. [41], CCN51 beans are recognized for their high bitterness, astringency, and acceptable taste.

**Figure 3:**
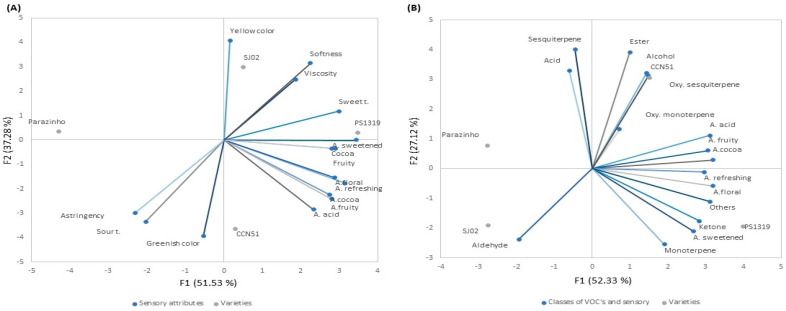
Principal component analysis (PCA) relating sensory attributes and cocoa honey varieties (**A**). PCA relating VOC classes, aroma sensory attributes, and cocoa honey varieties (**B**). A. Acid = aroma acid; A. Sweetened = aroma sweetened; A. Cocoa/Cocoa Pulp = aroma cocoa/cocoa pulp; A. Fruity = aroma fruity; A. Refreshing = aroma minty/refreshing; A. Floral = aroma floral; Sour t = sour taste; Sweet t = sweet taste; Cocoa = cocoa/cocoa pulp.

Variety PS1319 differed from the others in viscosity, while softness was more prevalent in varieties SJ02 and PS1319 compared to varieties CCN51 and Parazinho. Principal component analysis (PCA) was conducted to compare sensory attributes and cocoa honey varieties (Figure 3A). The PCA factors explained 51.53% and 37.28% of the data variation in the first two principal components, respectively.

The PCA in Figure 3A revealed that the PS1319 and SJ02 varieties exhibited similar profiles, characterized by yellowish color, higher viscosity, softness, and sweet taste. Conversely, the CCN51 and Parazinho varieties showed similar attributes, including a greenish color, and an acidic and astringent flavor. These results are supported by Table 2, which presents the sum of the rankings obtained through the RDA conducted by trained tasters. Additionally, the PCA indicated that the type of variety influenced the sensory attributes of cocoa honey. In their studies with cocoa liquor, Sukha et al. [42] concluded that cocoa variety strongly influenced the flavor potential.

According to the average scores attributed by consumers in the acceptance test (Table 3), a significant difference (*p* < 0.05) was observed only for the attributes of flavor and overall quality among the four varieties of cocoa honey studied. Flavor is one of the most important quality attributes, influencing the acceptability of cocoa products, such as cocoa honey. All varieties showed good sensory acceptability, with hedonic scale scores ranging from, “I liked it slightly” to “I liked it moderately”. Varieties SJ02 and CCN51 obtained higher scores for the flavor attribute, without differences between them, corresponding to, “I liked it moderately”, and differing significantly (*p* < 0.05) from PS1319 and Parazinho varieties. This difference could possibly be attributed to the presence of bitterness and astringent flavors. Table 2 indicates that the Parazinho variety had the highest content of astringency and the lowest sweet taste, while the SJ02 variety was considered to have the highest sweet taste. However, regarding overall quality, both the SJ02 and CCN51 varieties also obtained the highest scores, ranging from, “I liked it slightly” to “I liked it moderately”, but they did not differ significantly (*p* > 0.05) from each other.

In terms of purchase intention, the SJ02 and CCN51 varieties showed the highest results, with a score of “probably would buy”, while the Parazinho variety had the lowest purchase intention (“maybe I would buy/maybe I would not buy”). As discussed earlier, these results can be explained by Table 2, where the RDA tasters indicated that the CCN51 and SJ02 varieties had higher levels of flavor attributes and more astringency, while they attributed less sweetness to Parazinho. A similar result was obtained by Andrade et al. [19] when evaluating five samples of chocolate using doughs with different fermented cocoa contents.

### 2.5. Correlation of Sensory Analysis with VOCs

The principal component analysis (PCA) shown in Figure 3B correlates the sensory attributes of aroma assessed in the RDA with the VOCs and the four cocoa varieties studied. The PCA factors explained 52.33% and 27.12% of the data variability in the first two principal components (PC1 and PC2). VOCs were categorized into classes, namely, acids, alcohols, aldehydes, esters, ketones, monoterpenes, oxygenated monoterpenes, sesquiterpenes, oxygenated sesquiterpenes, and others.

Figure 3B reveals that the CCN51 variety was rich in VOCs, predominantly composed of acids, sesquiterpenes, esters, oxygenated sesquiterpenes, and oxygenated monoterpenes, exhibiting sensory attributes such as acidic, fruity, cocoa/cocoa pulp, and minty/refreshing aroma. In samples of cocoa liquor of the CCN51 variety, according to Rojas et al. [20], the main types of compounds identified were aldehydes, ketones, alcohols, pyrazines, esters, terpenes, acids, and lactones. The PS1319 variety demonstrated a predominance of ketones, monoterpenes, and other volatile organic compounds with a refreshing/minty, floral, and sweet aroma. According to Rodrigues-Campos et al. [26], some ketones are known to impart floral notes to cocoa. The SJ02 variety exhibited a predominance of aldehydes, while Parazinho stood out for the presence of acids and sesquiterpenes, lacking aroma descriptors.

The aroma composition of cocoa products is closely linked to the distinct post-harvest processing conditions, as well as the variety and origin of the cocoa itself [43]. High-quality “cocoa” is mainly produced from Criollo or Trinitario varieties, which typically feature fruity, floral, herbal, woody, and caramel notes, while common cocoa beans are sourced from Forastero variety [44]. Since CCN51, PS1319, and SJ02 are hybrids resulting from the genetic enhancement of Trinitario cacao, unlike Parazinho, which belongs to the Forastero type and has not undergone genetic improvement [3], the results obtained in the PCA (Figure 3B) indicated that the CCN51 and PS1319 varieties obtained the highest abundances of VOCs and aroma descriptors. This may be attributed to the origin of these varieties in Trinitario cocoa, renowned for its high quality.

## 3. Materials and Methods

### 3.1. Standards and Materials

Standard solutions of n-alkanes C_7_–C_30_, nonanoic acid, dodecanoic acid, tetradecanoic acid, pentadecanoic acid, palmitic acid, 1-heptanol, 1-dodecanol, 1-hexadecanol, benzaldehyde, octanal, (*E*)-2-octenal, 2-ethylhexyl salicylate, 2-heptanona, geranyl acetone, D-limonene, and linalool were acquired from Sigma–Aldrich (Burlington, MA, USA). All standards used in GC-MS analysis were of ≥95% purity.

SPME fibers, divinylbenzene/carboxen/polydimethylsiloxane (DVB/CAR/PDMS, 65 μm), polydimethylsiloxane/divinylbenzene (PDMS/DVB, 50/30 μm), carboxen/polydimethylsiloxane (CAR/PDMS, 75 μm), and polyacrylate (PA, 85 μm), used in this study to extract the VOCs from the samples, and the SPME holder for manual sampling, were purchased from Supelco (Bellefonte, PA, USA).

### 3.2. Obtaining Cocoa Honey

Cocoa honey was obtained from ripe fruits of the CCN51, PS1319, SJ02, and Parazinho varieties harvested in the city of Presidente Tancredo Neves (Bahia, Brazil; latitude: 13°27′14″ south; Longitude: 39°25′15″ west), in May 2022. Cocoa honey was collected before cocoa bean fermentation. Initially, a pre-selection was carried out, discarding unripe, damaged, and advanced senescent fruits. The almonds were placed in a manual stainless-steel cold press to obtain cocoa honey. Finally, the cocoa honey was stored in polyethylene bottles and kept at −18 °C (±0.5 °C) until the beginning of the analyses. Three batches of cocoa honey were obtained for each variety.

### 3.3. HS-SPME Method

An amount of 10 mL of cocoa honey was placed in sealed 20 mL glass vials for extracting VOCs through headspace solid-phase microextraction (HS-SPME). Extraction was performed by placing the flask in an aluminum heating block (4 cm high by 14 cm in diameter) on a temperature-controlled heating plate [45]. SPME extractions were performed from the headspace of the samples, after optimization, according to the following conditions: DVB/CAR/PDMS fiber (65 µm), equilibration time of 20 min, extraction time of 78 min, extraction temperature of 85 °C, and stirring speed of 171 rpm, using a magnetic bar. After extracting and pre-concentrating VOCs, the fiber was inserted directly into the GC injector for 3 min.

### 3.4. Optimization of the HS-SPME Conditions

A mixture containing 25% of each of the four varieties of cocoa honey was used to optimize the extraction method by HS-SPME. As an initial step, a screening 2^4^ full factorial design [46] was performed to evaluate significant variables involved in HS-SPME. Three replications were performed at the central point of the factorial design to quantify the experimental error. The variables evaluated by the screening experimental design were the extraction temperature, the extraction time, the equilibrating time, and the stirring speed. The levels employed in these experiments are listed in Table S1. The response evaluated during all experiments was the total sum of peak areas obtained in the GC-MS analysis.

Once significant variables were obtained, a central composite design, with 19 experiments and 5 replicates in central points, and response surface methodology [46] were carried out to locate the optimum values of temperature, extraction time, and stirring speed. The experimental levels involved in central composite design optimization are listed in Table S2. The statistical experimental design and optimization calculations were performed using the Statistica 7.0 software (Statsoft, Tulsa, OK, USA).

### 3.5. GC-MS Analysis

The volatile organic compounds were analyzed using a gas chromatograph coupled with a mass spectrometer (GC-MS; Model QP2010 Plus, Shimadzu^®^, Kyoto, Japan), using a capillary column model HP-5MS (5%—phenylmethylpolysiloxane, 30.0 m × 0.25 mm I.D × 0.25 μm, Restek^®^, Bellefonte, PA, USA). Cocoa honey compounds were determined on three batches of samples of each variety, with each batch evaluated in triplicate (totaling nine analyses for each variety).

The sample analysis method followed the parameters: oven temperature at 30 °C (held for 2 min), heating from 0.5 °C min^−1^ to 38 °C (for 2 min), followed by heating from 4.0 °C min^−1^ to 90 °C (hold 2 min), heating from 4 °C min^−1^ to 200 °C (hold 2 min), heating from 10 °C min^−1^ to 280 °C, and heating from 30 °C min^−1^ to 300 °C, totaling a run of 73.17 min. The injector temperature was 250 °C, splitless mode was used, and flow rate of 0.61 mL min^−1^. Helium (99.999%) was used as a carrier gas. The MS conditions were as follows: transfer line temperature of 250 °C, ion source temperature of 250 °C, and employing electron impact ionization at 70 eV.

VOCs were identified by comparing the obtained retention times and mass spectra with those of the pure analytical standards. Mass spectra were also compared to the data system library (NIST 147 Database). The linear retention index (LRI) was determined using a homologous series of C_7_–C_30_ n-alkanes and the results were compared with values reported in the literature for similar chromatographic columns. The percentage of individual peaks was obtained by normalizing the measured peak area without correction factors.

### 3.6. Multivariate Data Analysis

A data matrix containing the areas of identified peaks was subjected to principal component analysis (PCA) and hierarchical cluster analysis (HCA) with pre-treatment by autoscaling [46]. As the PCA is a well-known multivariate analysis chemometric technique, which facilitates the visualization of clustering trends, using all the information contained in many variables, this tool was used to intensify the clustering trends of different varieties of honey from cocoa, based on their VOC profiles. HCA is a statistical tool for grouping samples based on similarity, measuring distances between all possible pairs of samples in dimensional space.

### 3.7. Sensory Evaluation of Cocoa Honey

Initially, the project was approved by the Research Ethics Committee of the Faculty of Pharmacy of the Federal University of Bahia (UFBA; CAAE: 65163322.9.0000.8035).

#### 3.7.1. Microbiological Analysis

Before carrying out the sensory evaluation, microbiological analyses were carried out for *Salmonella* spp., *Escherichia coli*, and molds and yeasts, in compliance with Normative Instruction No. 161 [47] through the methodology of Silva et al. [48].

#### 3.7.2. Ranking Descriptive Analysis (RDA)

Analyses were performed at the Sensory Analysis Laboratory of the Faculty of Pharmacy at the Federal University of Bahia (UFBA). The method used for the sensory characterization was the ranking descriptive analysis (RDA), an adaptation of the quantitative descriptive analysis (QDA), which describes the main characteristics that make up the appearance, aroma, flavor, and texture of food, in addition to measuring the intensity of perceived sensations. Ranking descriptive analysis (RDA) was proposed by Richter et al. [15], where the evaluation of products is performed by ordering the intensity of the evaluated attributes.

Here, 23 previously trained tasters, aged 18 to 45, evaluated the cocoa honey samples in a single session. In the first training session, the evaluators were subjected to a basic recognition test of the four basic tastes (sweet, sour, bitter, and salty) and ten aromas. In the second session, they underwent the triangular test to identify bitter, acidic, and sweet tastes. In the third session, they were presented with the paired comparison test to identify acidic and bitter tastes. In the fourth session, they underwent the ordering test to order the sweet, bitter, and acidic tastes in increasing order of intensity, in addition to the test to order the sweet, bitter, acidic, and fruity aromas in an increasing order. In the fifth session, the network method was applied. Tasters received the four samples in pairs (A and B; C and D) and were asked to describe their similarities and differences in appearance, aroma, flavor, and texture, followed by a group discussion after the survey of attributes. In the sixth session, the tasters were presented with the RDA test form and the reference table (Table S4).

The seventh session was the final evaluation, where 40 mL of each sample was served in 50 mL white disposable cups coded with three-digit random numbers and randomized between tasters and repetitions, in individual booths illuminated by white light. Tasters were instructed to cleanse their palates by drinking water and chewing a biscuit. The four samples were ordered in ascending order of the generated attributes, obtaining the ordering totals according to Table 2. Three tasters were excluded from the data analysis due to incorrectly filling out the evaluation form. All samples were evaluated in triplicate during the session. The data collected referring to all attributes were evaluated using the Friedman test, at a 5% probability level.

#### 3.7.3. Acceptance

One hundred and eight consumers carried out the acceptance test in a single session. The cocoa honey varieties were served in white disposable cups, coded with three-digit random numbers, containing 10 mL of the samples at 4 ± 1 °C, in individual booths illuminated by white light. Consumers evaluated the appearance, aroma, flavor, consistency, and overall quality attributes, using a 9-point structured hedonic scale (1—“I disliked it extremely” to 9—“I liked it extremely”), in addition to a purchase intention scale (1—“certainly would not buy” to 5—“certainly would buy”). In addition, a questionnaire was applied to assess the consumer profile. 

### 3.8. Statistical Analysis

For VOC data, the MetaboAnalyst 5.0 program was used. Sensory profile results were expressed as mean ± standard deviation and statistical analysis was performed using the statistical software XLStat version 7.8. An analysis of variance (ANOVA) followed by the Tukey test was performed to determine statistically significant differences between means (*p* < 0.05).

## 4. Conclusions

The optimized HS-SPME/GC-MS method enhanced the extraction efficiency, identifying 84 VOCs in cocoa honey from 4 varieties. Sensory analysis revealed that PS1319 and SJ02 had similar profiles, with yellowish color, higher viscosity, softness, and sweet taste. The study concluded that the cocoa honey variety influenced VOC concentrations and sensory profiles. SJ02 and CCN51 showed the highest acceptability and purchase intention, highlighting their potential as promising sources for the food industry. Further research is required to identify potential quality markers among these VOCs for cocoa honey produced from different cocoa tree varieties.

## Figures and Tables

**Table 2:** Sum of orders (order totals) of sensory attributes for each variety of cocoa honey.

Attributes	Varieties				
	CCN51	PS1319	SJ02	Parazinho	Vcritical
Appearance					
Yellow color	80 ^a^	164 ^b^	199 ^c^	157 ^b^	22
Greenish color	202 ^a^	131 ^b^	120 ^b^	147 ^c^	22
Aroma					
Acid	169 ^a^	172 ^a^	128 ^b^	131 ^b^	22
Sweetened	149 ^a^	187 ^b^	149 ^a^	115 ^c^	22
Cocoa/cocoa pulp	168 ^a^	182 ^a^	128 ^b^	123 ^b^	22
Fruity	169 ^a^	172 ^a^	135 ^b^	124 ^b^	22
Minty/refreshing	166 ^a^	174 ^a^	141 ^b^	119 ^b^	22
Floral	157 ^a^	181 ^b^	133 ^c^	129 ^c^	22
Flavor					
Sour taste	187 ^a^	123 ^b^	118 ^b^	172 ^a^	22
Sweet taste	150 ^a^	174 ^b^	191 ^b^	86 ^c^	22
Cocoa/cocoa pulp	164 ^a^	156 ^a^	165 ^a^	115 ^b^	22
Fruity	166 ^a^	159 ^a^	167 ^a^	107 ^b^	22
Astringency	168 ^a^	136 ^b^	125 ^b^	171 ^a^	22
Texture					
Viscosity	126 ^a^	176 ^b^	153 ^a^	145 ^a^	22
Softness	129 ^a^	167 ^b^	171 ^b^	133 ^a^	22

Values followed by the same letter, in the same line, do not differ from each other by the Friedman test at the 5% probability level.

**Table 3:** Acceptance test and purchase intention averages for different varieties of cocoa honey.

Attributes	Varieties			
	CCN51	PS1319	SJ02	Parazinho
Sensory acceptance				
Appearance	6.6 ± 1.67 ^a^	6.63 ± 1.71 ^a^	6.75 ± 1.61 ^a^	6.66 ± 1.47 ^a^
Aroma	6.41 ± 1.66 ^a^	6.47 ± 1.84 ^a^	6.22 ± 1.63 ^a^	6.31 ± 1.62 ^a^
Flavor	7.11 ± 1.59 ^a^	6.95 ± 1.98 ^ab^	7.13 ± 1.89 ^a^	6.42 ± 1.92 ^b^
Consistency	6.88 ± 1.77 ^a^	7.02± 1.72 ^a^	6.94 ± 1.82 ^a^	6.52 ± 1.90 ^a^
Overall quality	7.08 ± 1.55 ^ab^	6.93 ± 1.84 ^ab^	7.21 ± 1.52 ^a^	6.53 ± 1.71 ^b^
Purchase intention				
	3.56 ± 1.17 ^a^	3.39 ± 1.36 ^ab^	3.71 ± 1.23 ^a^	2.99 ± 1.27 ^b^

Means followed by the same letter on the line do not differ significantly from each other (Tukey test; *p* < 0.05).

## Data Availability

The data presented in this study are available in the article and Supplementary Materials.

## Supplementary Materials

The following supporting information can be downloaded at: https://www.mdpi.com/article/10.3390/molecules29133194/s1. Table S1: Variable levels employed for screening of 2^4^ full factorial optimization of the HS-SPME method of cocoa honey. Table S2: Variable levels employed for central composite design from optimization of the HS-SPME method of cocoa honey. Table S3: Experiments carried out to confirm HS-SPME optimal conditions estimated by response surface methodology at extrapolation conditions (n = 3). Table S4: Descriptive terms and reference materials.

## References

[1] International Cocoa Organization (ICCO) (2024). Quarterly Bulletin of Cocoa Statistics, Vol. L, No. 1, Cocoa Year 2023/24..

[2] Instituto Brasileiro de Geografia e Estatística (IBGE) (2023). Levantamento Sistemático da Produção Agrícola. IBGE/LSPA. Tabela 6588—Série Histórica da Estimativa Anual da Área Plantada, Área Colhida, Produção e Rendimento Médio dos Produtos Agrícolas.

[3] Serra W.S., Sodré G.A. Manual do Cacauicultor: Perguntas e Respostas. Brasil. Ilhéus, BA, CEPLAC/CEPEC. Boletim Técnico, 2021, nº 221. 190p. https://www.gov.br/agricultura/pt-br/assuntos/ceplac/publicacoes/boletins-tecnicos-bahia/boletim-tecnico-no-221-2021_compressed.pdf.

[4] Hegmann E., Niether W., Phillips W., Rohsius C., Lieberei R. (2020). Besides variety, also season and ripening stage have a major influence on fruit pulp aroma of cacao (*Theobroma cacao* L.). J. Appl. Bot. Food Qual..

[5] Guirlanda C.P., Silva G.G., Takahashi J.A. (2021). Cocoa honey: Agroindustrial waste or underutilized cocoa by-product?. Future Foods.

[6] Santos C.O., Bispo E.S., Santana L.R.R., Carvalho R.D.S. (2014). Use of “cocoa honey” (*Theobroma cacao* L.) for diet jelly preparation: An alternative technology. Rev. Bras. Frutic..

[7] Freitas R.V.S., Silva F.L.H., Cavalcante J.A., Costa I.I.S., Sarmento D.H.A., Braga R.C., Silva F.S., Barbosa M.C.F., Rodrigues E.A. (2022). Evaluation of nutritional composition, characterization and correlation of pulp quality parameters of cocoa. Res. Soc. Dev..

[8] Puerari C., Magalhães K.T., Schwan R.F. (2012). New cocoa pulp-based kefir beverages: Microbiological, chemical composition and sensory analysis. Food Res. Int..

[9] Neto B.A.M., Carvalho E.A., Pontes K.V., Barretto W.S., Sacramento C.K. (2013). Chemical, physico-chemical and sensory characterization of mixed açai (*Euterpe oleracea*) and cocoa’s honey (*Theobroma cacao*) jellies. Rev. Bras. Frutic..

[10] Yulianaa N., Nurainya F., Sari G.W., Sumardi, Widiastuti E.L. (2023). Total microbe, physicochemical property, and antioxidative activity during fermentation of cocoa honey into kombucha functional drink. Appl. Food Res..

[11] Lannes S.C.D.S., Silva M.V., Silva E.N., Ramos D.D.C., Su F. (2013). Food Compositions of Chocolate and Edible Ice Cream Containing Cocoa Honey University of São Paulo (USP, Sao Paulo, Brazil) and State University of Southwest Bahia (UESB, Bahia, Brazil). https://patents.google.com/patent/BR102013005053B1.

[12] Rodrigues L.B.O., Dias J.C.T., Uetanabaro A.P.T., Bonomo P. (2019). Produção de Cerveja Artesanal Utilizando Mel de Cacau Como Adjunto. https://patents.google.com/patent/BR102019008742A2/pt?oq=BR+102019008742.

[13] Mohammadi A., Yamini Y., Alizadeh N. (2005). Dodecylsulfate-doped polypyrrole film prepared by electrochemical fiber coating technique for headspace solid-phase microextraction of polycyclic aromatic hydrocarbons. J. Chromatogr. A..

[14] Dutcosky S.D. (2019). Análise Sensorial de Alimentos.

[15] Richter V.B., Almeida T.C.A., Prudencio S.H., Benassi M.T. (2010). Proposing a ranking descriptive sensory method. Food Qual. Prefer..

[16] Silva R.C.S.N., Minim V.P.R., Carneiro J.D.S., Nascimento M., Lucia S.M.D., Minim L.A. (2013). Quantitative sensory description using the optimized descriptive profile: Comparison with conventional and alternative methods for evaluation of chocolate. Food Qual. Prefer..

[17] Brokl M., Bishop L., Wright C.G., Liu C., McAdam K., Focant J.F. (2014). Multivariate analysis of mainstream tobacco smoke particulate phase by headspace solid-phase microextraction coupled with comprehensive two-dimensional gas chromatography–time-of-flight mass spectrometry. J. Chromatogr. A.

[18] Valdeci S.B., Thais M.U., Neyde A.B., Claudia M.R., Vânia M.F.P., Eduardo M.D.A. (2019). Dynamics of volatile compounds in TSH 565 cocoa clone fermentation and their role on chocolate flavor in Southeast Brazil. J. Food Sci. Technol..

[19] Andrade A.B., Cruz M.L., Oliveira F.A.S., Soares S.E., Druzian J.I., Santana L.R., Souza C.O., Bispo E.S. (2021). Influence of under-fermented cocoa mass in chocolate production: Sensory acceptance and volatile profile characterization during the processing. LWT-Food Sci. Technol..

[20] Rojas E.O., Rúales F.H., Perdomo D.A., Mora J.P.J. (2022). Evaluación del método de extracción SPME-GC-MS para el análisis de compuestos orgánicos volátiles en licor de cacao de Nariño-Colombia. Rev. ION.

[21] Torres-Moreno M., Torrescasana E., Salas-Salvadó J., Blanch C. (2015). Nutritional composition and fatty acids profile in cocoa beans and chocolates with different geographical origin and processing conditions. Food Chem..

[22] Haase T.B., Schweiggert-Weisz U., Ortner E., Zorn H., Naumann S. (2021). Aroma Properties of Cocoa Fruit Pulp from Different Origins. Molecules.

[23] Pino J.A., Ceballos L., Quijano C.E. (2010). Headspace Volatiles of *Theobroma cacao* L. Pulp from Colombia. J. Essent. Oil Res..

[24] Ascrizzi R., Flamini G., Tessieri C., Pistelli L. (2017). From the raw seed to chocolate: Volatile profile of blanco de Criollo in different phases of the processing chain. Microchem. J..

[25] Chetschik I., Kneubul M., Chatelain K., Schluter A., Bernath K., Huhn T. (2017). Investigations on the Aroma of Cocoa Pulp (*Theobroma cacao* L.) and Its Influence on the Odor of Fermented Cocoa Beans. J. Agric. Food Chem..

[26] Rodriguez-Campos J., Escalona-Buendía H.B., Orozco-Avila I., Lugo-Cervantes E., Jaramillo-Flores M.E. (2011). Dynamics of volatile and non-volatile compounds in cocoa (*Theobroma cacao* L.) during fermentation and drying processes using principal components analysis. Food Res. Int..

[27] Utrilla-Vázquez M., Rodríguez-Campos J., Avendaño-Arazate C.H., Gschaedler A., Lugo-Cervantes E. (2020). Analysis of volatile compounds of five varieties of Maya cocoa during fermentation and drying processes by Venn diagram and PCA. Food Res. Int..

[28] Miyazawa M., Hashidume S., Takahashi T., Kikuchi T. (2012). Aroma evaluation of Gamazumi (*Viburnum dilatatum*) by aroma extract dilution analysis and odour activity value. Phytochem. Anal..

[29] Velasquez-Reyes D., Gschaedler A., Kirchmayr M., Avendano-Arrazate C., Rodríguez-Campos J., Calva-Estrada S.J., Lugo-Cervantes E. (2021). Cocoa bean turning as a method for redirecting the aroma compound profile in artisanal cocoa fermentation. Heliyon.

[30] Rottiers H., Sosa D.A.T., Winne A., Ruales J., Clippeleer J., Leersnyder I., Wever J., Everaert H., Messens K., Dewettinck K. (2019). Dynamics of volatile compounds and flavor precursors during spontaneous fermentation of fine flavor Trinitario cocoa beans. Eur. Food Res. Technol..

[31] Aprotosoaie A.C., Luca S.V., Miron A. (2016). Flavor Chemistry of Cocoa and Cocoa Products—An Overview. Compr. Rev. Food Sci. Food Saf..

[32] Zhang Z., Li G. (2010). A review of advances and new developments in the analysis of biological volatile organic compounds. Microchem. J..

[33] Bryant R.J., McClung A.M. (2011). Volatile profiles of aromatic and non-aromatic rice cultivars using SPME/GC–MS. Food Chem..

[34] Owusu M., Petersen M.A., Heimdal H. (2012). Effect of fermentation method, roasting and conching conditions on the aroma volatiles of dark chocolate. J. Food Process. Preserv..

[35] Janek K., Niewienda A., Wöstemeyer J., Voigt J. (2016). The cleavage specificity of the aspartic protease of cocoa beans involved in the generation of the cocoa-specific aroma precursors. Food Chem..

[36] Kamal-Eldin A., Andersson R. (1997). A multivariate study of the correlation between tocopherol content and fatty acid composition in vegetable oils. J Am Oil Chem Soc..

[37] Calva-Estrada S.J., Utrilla-Vázquez M., Vallejo-Cardona A., Roblero-Pérez D.B., Lugo-Cervantes E. (2020). Thermal properties and volatile compounds profile of commercial dark chocolates from different genotypes of cocoa beans (*Theobroma cacao* L.) from Latin America. Food Res. Int..

[38] Nascimento M.B., Souza T.L., Maia D.L.S., Amorim L.R., Ribeiro A.S.L., Mamede M.E.O., Maciel L.F., Santos Júnior A.F., Mesquita P.R.R., Soares S.E. (2024). Determination of Mineral Profile Using MIP OES and Physicochemical Composition of Cocoa Honey from Different Cocoa Varieties (*Theobroma cacao* L.). Food Anal. Methods.

[39] Papalexandratou Z., Nielsen D.S. (2016). It’s gettin’ hot in here: Breeding robust yeast starter cultures for cocoa fermentation. Trends Microbiol..

[40] Eskes A.B., Guarda D., Garcia L., Garcia P. (2007). Is genetic variation for sensory traits of cocoa pulp related to fine flavor cocoa traits?. INGENIC Newsl..

[41] Boza E.J., Motamayor J.C., Amores F., Cedeno-Amador S., Tondo C.L., Livingstone D.S., Schnell R., Gutiérrez O. (2014). Genetic characterization of the cacao cultivar CCN 51: Its impact and significance on global cacao improvement and production. J. Am. Soc. Hortic. Sci..

[42] Sukha D.A., Butler D.R., Umaharan P.E., Boulton E. (2008). The use of an optimised organoleptic assessment protocol to describe and quantify different flavour attributes of cocoa liquors made from Ghana and Trinitario beans. Eur. J. Food Sci. Technol..

[43] Kongor J.E., Hinneh M., Van deWalle D., Afoakwa E.O., Boeckx P., Dewettinck K. (2016). Factors influencing quality variation in cocoa (*Theobroma cacao*) bean flavour profile—A review. Food Res. Int..

[44] ICCO (2023). Fine or Flavour Cocoa. https://www.icco.org/fine-or-flavor-cocoa/.

[45] Mesquita P.R.R., Nunes. E.C., Santos F.N., Bastos L.P., Costa M.A.P.C., Rodrigues F.M., Andrade J.B. (2017). Discrimination of *Eugenia uniflora* L. biotypes based on volatile compounds in leaves using HS-SPME/GC–MS and chemometric analysis. Microchem. J..

[46] Brereton R.G. (2003). Chemometrics: Data Analysis for the Laboratory and Chemical Plant.

[47] ANVISA (2022). Instrução Normativa—IN Nº 161. ANEXO I: Padrões Microbiológicos para Alimentos, Com Exceção dos Alimentos Comercialmente Estéreis. http://antigo.anvisa.gov.br/documents/10181/2718376/IN_161_2022_.pdf/b08d70cb-add6-47e3-a5d3-fa317c2d54b2.

[48] Silva N., Junqueira V.C.A., Silveira N.F.A., Taniwaki M.H., Gomes R.A.R.G., Okazaki M.M. (2021). Manual de Métodos de Análise Microbiológica de Alimentos e Água.

