# Supplementary material for: Optimization of HS-SPME/GC-MS Method for Determining Volatile Organic Compounds and Sensory Profile in Cocoa Honey from Different Cocoa Varieties (Theobroma cacao L.)
Source: Molecules. 2024 Jul 5;29(13):3194. doi: 10.3390/molecules29133194 (PMC11243235; doi:10.3390/molecules29133194)
Supplement: Supplementary file 1 [file molecules-29-03194-s001.zip › molecules-3064802-supplementary.pdf]

Supplementary material

Optimization of HS-SPME/GC-MS Method for Determining Volatile Organic Compounds and Sensory Profile in Cocoa Honey from Different Cocoa Varieties (*Theobroma cacao* L.)

Table S1. Variables levels employed for screening of 2^4 full factorial optimization of HS-SPME method of cocoa honey.

Variable	Coded Variable		
	(-1)	(0)	(+1)
Extraction temperature, ºC	40	60	80
Extraction time, min	10	20	30
Stirring speed, rpm	100	250	400
Equilibrium time, min	0	10	20

Table S2. Variables levels employed for central composite design from optimization of HS-SPME method of cocoa honey.

Variable	Coded Variable				
	(-1.68)	(-1)	(0)	(+1)	(+1.68)
Extraction temperature, ºC	73	80	90	100	107
Extraction time, min	13	50	60	70	77
Stirring speed, rpm	230	300	400	500	570

Table S3. Experiments carried out to confirm HS-SPME optimal conditions estimated by response surface methodology at extrapolation conditions (n=3).

Temperature extraction/ ºC	Time extraction/min	Stirring speed /rpm	Response
85	78	171	4.07 $x10^8$
85	68	161	3.93 $x10^8$
85	88	181	2.44 $x10^8$
95	78	171	3.25 $x10^8$
95	88	181	3.35 $x10^8$
75	68	161	3.74 $x10^8$

Table S4. Descriptive terms and reference materials.

Sensory Descriptors	Definition	Reference Material
**Appearance**		
Yellow color	Has a light shade of yellow	**Less intense**: Cocoa pulp + 20 drops orange juice (SL).<br>**More intense**: Cocoa pulp + 10% SL.
Greenish color	A yellow tint that tends slightly to green. Similar to lime green	**Less**: Cocoa pulp + 20 drops lime juice.<br>**More**: Persian lime juice.
**Aroma**		
Acid	Odor relative to acetic acid.	**Less**: Cocoa pulp + 1% apple cider vinegar.<br>**More**: Cocoa pulp + 5% apple cider vinegar.
Sweet	Odor obtained from sugar/caramel	**Less:** Cocoa pulp.<br>**More**: Cocoa pulp + 10% sugar heated to 120oC to brown color.
Cocoa/Cocoa pulp	Odour relating to cocoa/chocolate pulp	**Less**: Cocoa pulp + 10% nibs of crushed cocoa.<br>**More**: Cocoa pulp + 40% crushed cocoa nibs.

Fruity	Odor obtained from tangerine, lemon and other citrus fruits	**Less:** Cocoa pulp + 5% tangerine, orange, lemon (TLL) solution.<br>**More:** Cocoa pulp + 20% TLL
Minty/Refreshing	Mint odor. Feeling of freshness of the mint.	**Less**: Cocoa pulp + 3% solution of aromatic herbs of mint (EM).<br>**More**: Cocoa pulp + 10% EM.
Floral	Sensation that refers to the floral-fruity odor (flowers: lavender, roses, jasmine, magnolias).	**Less:** Cocoa pulp + 5% floral solution (LRJ).<br>**More:** Cocoa pulp + 30% LRJ.
**Flavor**		
Acidic taste	I like citric acid.	**Less**: Cocoa pulp + 0.05% citric acid.<br>**More**: Cocoa pulp + 1.% citric acid.
Sweet taste	Flavor associated with the presence of sugars. Sweet caramel flavor.	**Less**: Cocoa pulp + 1.0% sugar solution.<br>**More**: Cocoa pulp + 10.0% sugar solution
Cocoa/Cocoa pulp	Flavor associated with cocoa pulp/cocoa *nibs*.	**Less**: Cocoa pulp + 10% nibs of crushed cocoa.<br>**More**: Cocoa pulp + 40% nibs of crushed cocoa.
Fruity	Flavor relative to citrus fruits (tangerine, lemon, orange)	**Less**: Cocoa pulp + 5% tangerine, orange, lemon (TLL) solution.<br>**More**: Cocoa pulp + 20% TLL solution.
Astringency	Dry sensation in the mouth when consuming unripe fruits. Sensation resulting from the contraction of the mucous membrane of the mouth.	**Less:** Cocoa pulp.<br>**More**: Cocoa pulp + green banana pulp.
**Texture/Sensation in the mouth**		
Viscosity	Resistance property to flow.	**Minus**: Cocoa pulp (1:1).<br>**More**: Cocoa pulp.

Softness	Feeling of silkiness in the mouth. Texture with a feeling of softness	**Less**: Cocoa pulp + 2% polyalcohol solution (PAL).<br>**More**: Cocoa pulp + 10% PAL.
